# Women are considerably more exposed to intimate partner violence than men in Rwanda: results from a population-based, cross-sectional study

**DOI:** 10.1186/1472-6874-14-99

**Published:** 2014-08-26

**Authors:** Aline Umubyeyi, Ingrid Mogren, Joseph Ntaganira, Gunilla Krantz

**Affiliations:** 1 Department of Epidemiology and Biostatistics, School of Public Health, College of Medicine and Health Sciences, University of Rwanda, P.O Box 5229, Kigali, Rwanda; 2 Department of Clinical Sciences, Obstetrics and Gynecology, Umea University, Förvaltningshuset, Universitetstorget 16, 901 87 Umea, Sweden; 3 Department of Public Health and Community Medicine, Sahlgrenska Academy at Gothenburg University, Gothenburg, Sweden

**Keywords:** Intimate partner violence, Rwanda, Prevalence, Risk factors, Exposure, Young men and women

## Abstract

**Background:**

Intimate partner violence (IPV) against women is an important, yet often neglected public health issue. The existence of gender norms imbalance expressed by men’s and women’s attitudes in relation to power and decision-making in intimate relationships may influence the magnitude of IPV. The aim of this study was to investigate the prevalence and potential risk factors of physical, sexual and psychological IPV in young men and women in Rwanda.

**Methods:**

This population-based, cross-sectional study included a representative sample of men and women from the Southern Province of Rwanda. Face-to-face interviews were performed using the World Health Organization (WHO) questionnaire for violence exposure to estimate past year and earlier in life IPV occurrence. Risk factor patterns were analyzed by use of bi- and multivariate logistic regression.

**Results:**

Women were, to a considerably higher extent, exposed to physical, sexual and psychological IPV than men. Of the women, 18.8% (n = 78) reported physical abuse in the past year, compared to 4.3% (n = 18) of men. The corresponding figures for women and men for sexual abuse were 17.4% (n = 71) and 1.5% (n = 6), respectively, and for psychological abuse, the corresponding figures were 21.4% (n = 92) and 7.3% (n = 32). Findings illustrate that violence against women was recurrent, as the highest frequency (>3 times) dominated in women for the various acts of all forms of violence. Identified risk factors for women’s exposure to physical violence were being low educated, having poor social support, being poor and having many children. For men exposed to physical violence, no statistically significant risk factor was identified.

**Conclusions:**

In this setting, IPV exposure was more common in women than men in the Southern Province of Rwanda. Promotion of gender equality at the individual level is needed to make a positive difference in a relatively short term perspective. Men’s lower reporting of IPV confirms women’s subordinate position, but men’s denial of incidents could also explain the gender role pattern.

## Background

Intimate partner violence (IPV) directed at women commonly occurs in all settings but with variations in prevalence and frequency [1]. However, only few studies present data on men’s exposure and these are mainly from high income countries [2,3]. In studies that include both men and women, a general finding is that men report less exposure to physical and sexual violence while psychological violence exposure is more evenly distributed, irrespective of time periods under investigation, i.e. past year or lifetime occurrence [3,4].

Studies from Sub-Saharan Africa on women’s exposure to IPV report past year exposure to physical and/or sexual violence at a range between 14 and 41% [5,6], but studies on both women’s and men’s exposure to IPV are rare. However, the 2011 Ugandan Demographic and Health Survey reports a high exposure rate of lifetime spousal physical violence for men and women (26% and 37% respectively) [7]. Important to note is that in studies in which both women and men are included, only women are asked about IPV exposure while men are regarded solely as perpetrators, with their personal characteristics analysed as tentative risk factors [8-10]. One study including both men and women failed to ask the men, but instead inquires women about both exposure and perpetration of IPV [11]. Findings reveal that women report being exposed to IPV to a considerably higher extent than their own perpetration of violence [11]. In addition, one study using data reported from seven countries in Eastern and Southern Sub-Saharan Africa with Rwanda included, confirms that women are more exposed to IPV than men [12].

Further, a number of studies from Sub-Saharan African countries have found that men and women justify wife-beating when the wife does not behave as expected, such as when arguing with the husband, neglecting the children, leaving home without informing their partner or refusing sex [8,13]. From Rwanda, the finding was that almost 50% of men and more than 60% of the women carried such attitudes [13]. This signifies that woman’s transgression of existing gender norms is considered an accepted reason for wife-beating by both women and men [13]. Important to remember, the gender norms and values in any society form an important part of the culture and are exemplified by men’s and women’s relationships and behaviour in everyday life. The power of men over women is deep-seated in all aspects of life and accepted as normal in most societies [14]. The Rwandan society is patriarchal society, where men occupy a dominant social position with control of resources and power while women are in a subordinate position [15]. Men are breadwinners and primarily working outside home while the woman carries the overall responsibility for the reproductive and the domestic work. The most problematic face of male domination is the use of violence against women.

Rwanda is a small, low income country located in central Africa with a population of 10.5 million inhabitants and a population density among the highest in Africa, 416 inhabitants/km^2^. In 2008, Rwanda adopted a law on the Prevention and Punishment of Gender-Based Violence (GBV) [16]. This includes all forms of violence, irrespective of whether exercised as so-called street violence or as IPV. The minimum penalty is a prison sentence of six months, while sexual abuse or rape that results in terminal illness or death of one’s spouse will lead to a sentence of life imprisonment.

Two studies conducted in Rwanda on IPV prevalence and its risk factors focused on pregnant women [17,18], in which past year prevalence of physical IPV is estimated to 35.1% [17]. Both studies report on acts such as hair pulling, slapping, choking, punching with fists, kicking and burning with a hot liquid [17,18]. Only one study explores globally physical IPV against men and women; but was conducted on a small sample of participants (16–51 years) enrolled in another quasi-experimental study. Nonetheless, it reports that 17% of the men and 29.7% of the women were victims of IPV in the past 3 months [19]. In conclusion, there is to date no study from Rwanda that investigates all forms of IPV in a representative population-based sample including both men and women.

The aim of this study was therefore to investigate the prevalence and frequency of physical, sexual and psychological intimate partner violence exposure in young men and women in Rwanda, and the risk factors associated with this exposure.

This study forms part of a project on violence and other traumatic episodes, mental health and barriers to care among young men and women, *The Rwandan Violence, Mental Health and Barriers to Care project (RwVMHBC-project)*.

## Methods

### Study design, study population and sample size

A cross-sectional study was conducted on a population representative sample of young adults, aged 20 to 35 years. The sample size was calculated based on an expected proportion of physical violence against women in the last 12 months equivalent to 20% [20], a desired level of absolute precision of 5% and an estimated design effect of 1.5. Accordingly, the study aimed to include 443 men and 443 women. The final sample consisted of 440 men and 477 women, with only two refusals to participate, which gave a response rate of 99.8%.

The study population was randomly selected from all the eight districts of the Southern province of Rwanda with a total population of 2.6 million people.

A multi-stage random sampling was used to identify households for inclusion. The selection of the primary sampling units (villages), households and study participants in the eight districts was made in three steps. Firstly, out of the total number of 3512 existing villages, 35 were randomly selected by using Epi-Info random function. Secondly, the number of households in each village was selected proportionate to the total number of households in each village. Lastly, the person to be interviewed was randomly selected among eligible people in each household, i.e. men and women aged between 20 and 35 years.

The first participant in each village was selected from the closest household to the center of the village. A calculated sampling interval in each village was applied to get the next household. If the first eligible participant was a female, the next was to be a male and the next a female then a male. Only one interview was conducted in each household for ethical reasons. If there was no eligible person living in the selected household, the closest household was approached. The rationale behind this procedure was that living conditions would be probably similar in a nearby household.

### Data collection procedures

A questionnaire was developed containing items on *physical violence*, *sexual violence* and *psychological abuse*. These items were selected from the Women’s health and life experiences questionnaire, a validated questionnaire developed by the WHO for research on IPV experience [21]. The questionnaire was translated into Kinyarwanda, the national language in Rwanda. This instrument has been shown to be cross-culturally valid [4,22,23] and was initially intended for detection of IPV against both men and women. To date, this instrument has only been used in one male population of the ten countries included in the WHO Multi-Country Study i.e. in Samoa [21]. Of the few published validation studies at hand, one was performed on men and women separately [4,23]. This study indicates that the dimensionality, i.e. the distribution of the included acts into the three dimensions physical, sexual and psychological violence respectively, assessed by principal components analysis with a promax rotation, is not well supported for men but is for women [4,23]. However, it additionally showed that items composing the three dimensions have good internal consistency for both men and women assessed by Cronbach alpha analyses [4,23].

The University of Rwanda, College of Medicine and Health Sciences, School of Public Health (SPH) was the lead implementer of the survey. A group of 13 experienced interviewers, clinical psychologists by training (composed of eight females and five males) and their two male supervisors were recruited. Two days of training was carried out followed by one day of questionnaire piloting. The data collection took place in the period between December 2011 - January 2012 and the data entry was performed by four skilled personnel from the SPH under the supervision of a data entry manager.

### Measures

#### Dependent variables

Violence occurrence was measured by exposure to physical violence (six items), sexual violence (three items) and psychological abuse (four items). The selected young men and women responded to exactly the same items. They were asked to indicate whether they had been exposed to any of the violent acts that their partner made against them either within the past 12 months or earlier in life. A three point scale was used to indicate the frequency of the various violent acts (from ‘once’ to ‘2-3’ times and ‘more than 3 times’) during the past year and earlier in life.

Summary measures were constructed for earlier in life and past year physical, sexual and psychological violence and finally dichotomised into any, as opposed to no violence experience. The respective reference categories were hereby composed of participants with no exposure to any of the forms of violence under investigation. Past year estimates of physical, sexual and psychological violence were selected for further analyses as they are less susceptible to recall bias compared to earlier in life figures [24].

#### Independent variables

Socio-demographic and psycho-social variables for the respondents and their partners were dichotomised and analysed as independent risk factors. *Age* was grouped into 2 categories (20–29 years and 30–35 years). *Number of children* was constructed with*,* zero to two children as the reference category and more than two children as the exposed category. E*ducational level* was grouped into incomplete primary and higher education (comprising of complete primary education and above or vocational training)*. Personal income per month* was grouped into earning 17,500Rwf or more per month as the reference category in comparison with earning less than 17,500Rwf (30US$) per month as exposed category. *Social support* was defined as having a friend or family member that would assist in case of illness, or would share food, share housing, lend money, assist with guidance when problems arise and offer support when in personal problems. A composite variable was constructed, dichotomised into good social support corresponding to having always, often or sometimes any of the six social support items, and poor social support equivalent to the absence of a positive response to any of the social support items.

*Living standards and assets available in the household* were used as a proxy of the socio-economic status. A *living standards* variable was constructed from the type of house, water source, electricity, cooking fuel and availability of a toilet facility. The various living standard items were merged and dichotomised into either improved living standard (having at least one of the living standard items in the reference category) or poor living standard (having none of the living standard items in the reference category) as the exposed category. In the same way, available *assets* in the household were inquired about: radio, television, refrigerator, bicycle, motorcycle, car, mobile phone and computer. These assets were merged and dichotomised into having at least one of the items (reference category) versus having none of the items (exposed category).

### Statistical analysis

Differences in socio-demographic factors between women and men were evaluated by the Pearson’s Chi-squared test for independence for all categorical variables. The violence exposure (n, %) and frequency of acts were used to indicate prevalence and perpetration of the various forms of violence. Risk factors were estimated by use of odds ratios (OR) with their 95% confidence interval (CI) in bi- and multivariate analyses to estimate predictors of past year exposure to physical, sexual or psychological violence. The multivariate analyses entered those variables that proved statistical significance in the bivariate analyses, one by one in a stepwise fashion, to control for possible confounding variables. Different models were used for different forms of violence. Hosmer-Lemeshow test was used to check the goodness fit of the final model. IBM SPSS Statistics vs. 20 was used for all statistical analyses.

This study has adhered to the STROBE guidelines on reporting of cross-sectional data Additional file 1.

### Ethical considerations

The research protocol and study tools were approved for scientific and ethical integrity by the Rwanda National Ethics Committee (Review Approval Notice No 165/RNEC/2011) and the National Institute of Statistics of Rwanda (No 1043/2011/10/NISR). The study strictly followed WHO guidelines on ethical issues related to violence research [25] i.e. all participants were informed about their free choice to participate and to withdraw at whatever time they wanted during the study. Interviewers secured written consent from all respondents before the interview. To maintain confidentiality, the interview was conducted in privacy and with only one interview in each household. Respondents were informed that questions could be sensitive and were reassured regarding the confidentiality of their responses.

Female respondents were exclusively interviewed by female interviewers and male respondents by male interviewers. No identifying information was entered into the dataset to secure anonymity. As IPV is a sensitive issue, which might induce strong feelings in those exposed, participants were informed that those in need of any kind of assistance could receive this at a nearby health centre that was informed beforehand about the study taking place.

## Results

### Socio-demographic and psycho-social characteristics

The study participants were aged 20 to 35 years and were almost equally distributed in different age-groups, the difference between the groups of men and women was threshold of statistical significance (p = .050). More women than men were married, 72% and 54%, respectively. Educational attainment was low in both men and women, but more men than women had completed primary school, 28.0% versus 18.6% (p = .006). The majority of the study participants were either unskilled or without any formal occupation (87.4% in men and 90.6% in women, p = .068) and most of them were earning less than 17.500 Rwandan Francs per month ( ~ 30 US$) (Table 1).

**Table 1:** Socio-demographic and psycho-social characteristics of the respondents and their partners

	***Total (N = 917)***		***Men (n = 440)***		***Women (n = 477)***		p-value*
**RESPONDENT CHARACTERISTICS**	***N***	**%**	***n***	**%**	***n***	**%**	
**Age groups** (n = 908)							.050
20-24	275	30.3	148	33.8	127	27.0	
25-29	300	33.0	144	32.9	156	33.2	
30-35	333	36.7	146	33.3	187	39.8	
**Marital status** (n = 912)							.000
Married or cohabiting	578	63.4	236	53.8	342	72.3	
Divorced or widowed	35	3.8	2	0.5	33	7.0	
Single	299	32.8	201	45.8	98	20.7	
**Number of children** (n = 915)							.000
No children	307	33.6	211	48.1	96	20.2	
1-3 children	467	51.0	192	43.7	275	57.8	
> 3 children	141	15.4	36	8.2	105	22.1	
**Level of education** (n = 768)							.006
Secondary school or university	117	15.2	50	13.3	67	17.0	
Complete primary or vocational training	178	23.2	105	28.0	73	18.6	
Incomplete primary school	473	61.6	220	58.7	253	64.4	
**Occupation** (n = 910)							.068
Civil servants	15	1.6	6	1.4	9	1.9	
Skilled workers or students	84	9.2	49	11.2	35	7.4	
Unskilled workers	512	56.3	230	52.5	282	59.7	
No formal occupation (subsist. farmer)	299	32.9	153	34.9	146	30.9	
**Personal income per month** (n = 912)							.005
More than 35,000 Rwf	30	3.3	19	4.3	11	2.3	
17,500-35,000 Rwf	55	6.0	36	8.2	19	4.0	
Less than 17,500 Rwf	827	90.7	382	87.4	445	93.7	
**Source of income** (n = 903)							.000
Salary	47	5.2	38	8.7	9	1.9	
Pension, disability grant or other	88	9.7	54	12.3	34	7.3	
No income	768	85.0	347	79.0	421	90.7	
**Social support** (n = 917)							.081
Improved	140	15.3	77	17.5	63	13.2	
Poor	777	84.7	363	82.5	414	86.8	
**PARTNER CHARACTERISTICS**							.000
**Partner’s age** (n = 577)							
20-24	97	16.8	67	28.2	30	8.8	
25-29	170	29.5	87	36.6	83	24.5	
30 and above	310	53.7	84	35.3	226	66.7	
**Partner’s educational level** (n = 475)							.412
Secondary school or university	55	11.6	21	10.0	34	12.8	
Complete primary or vocational training	141	29.7	59	28.1	82	30.9	
Incomplete primary school	279	58.7	130	61.9	149	56.2	
**Partner’s occupation** (n = 616)							.000
Civil servants	26	4.2	6	2.4	20	5.4	
Skilled worker or student	41	6.7	6	2.4	35	9.5	
Unskilled worker	342	55.5	120	48.6	222	60.2	
No formal occupation (Subsist. farmer)	207	33.6	115	46.6	92	24.9	
**HOUSEHOLD CHARACTERISTICS**							
**Household monthly income** (n = 883)							.566
17,500 or more	189	21.4	86	20.5	103	22.2	
< 17,500 Rwf	694	78.6	333	79.5	361	77.8	

* Chi square test for independence or Fisher’s exact probability test for difference between men and women.

### Living standards and assets in the household

As more than three quarter of the study participants and their partners were subsistence farmers, not employed, not earning an income, living standards and available assets in the household were used as a proxy for the socio-economic status. The majority had poor housing conditions, living in shacks or traditional dwellings with no electricity and no or inappropriate latrines and a large proportion of them used unsafe drinking water (Table 2). Even though an improved living standard was defined as possessing at least one item, an important proportion and mainly women were still in the poor living standard category, i.e. 36.1% of the women and 16.8% of the men, with no items indicating improved living standards. The same pattern was found for available assets in the household as a large proportion of male and female participants had none of the household assets explored, (males 28.7%; females 30.6%). To illustrate the spread of items in the living standard and assets variables, only 11.0% of the total population had three or more of the living standard items and the corresponding figure for assets in the household was 10.8%.

**Table 2:** Living standards and assets in the household

***Variables***	***Total (N = 917)***		***Men (n = 440)***		***Women (n = 477)***		P-value*
**Living standards**	N	%	n	%	n	%	
**Type of house** (n = 915)							
Combination of buildings, flat, maisonette, modern house	361	39.5	188	42.8	173	36.3	.050
Shack, traditional dwelling	554	60.5	251	57.2	303	63.7	
**Water source** (n = 912)							
Piped water, public tap, well/borehole,	544	59.6	338	77.0	206	43.6	.000
Surface water, tanker truck	368	40.4	101	23.0	267	56.4	
**Electricity** (n = 914)							
Yes	107	11.7	39	8.9	68	14.3	0.13
No	807	88.3	400	91.1	407	85.7	
**Cooking fuel** (n = 914)							.906
Kerosene, paraffin and other fuels	78	8.5	37	8.4	41	8.6	
Firewood and dung	836	91.5	403	91.6	433	91.4	
**Toilet facility** (n = 910)							
Flushed, improved latrine, other	24	2.6	14	3.2	10	2.1	.031
Latrine, no toilet	886	97.4	422	96.8	464	97.9	
**Summary measure living standards** (n = 917)							
Improved living standard (at least 1 item in the reference category of the living standard items)	671	73.2	366	83.2	305	63.9	.000
Low level of living standard (0 item in the reference category of the living standard items)	246	26.8	74	16.8	172	36.1	
**Assets in the household (HH)**							
Radio (n = 916)	586	64.0	300	68.2	286	60.1	.011
Television set (n = 916)	55	6.0	25	5.7	30	6.3	.781
Refrigerator (n = 915)	8	0.9	2	0.5	6	1.3	.290
Bicycle (n = 915)	145	15.8	87	19.8	58	12.2	.002
Motorcycle (n = 915)	17	1.9	4	0.9	13	2.7	.050
Car (n = 915)	12	1.3	3	0.7	9	1.9	.147
Mobile phone (n = 915)	282	30.8	122	27.8	160	33.6	.062
Computer (n = 914)	13	1.4	3	0.7	10	2.1	.094
**Summary variable for assets in the HH (n = 917)**							
Improved (at least one of 8 assets)	654	71.3	323	73.4	331	69.4	.189
Poor (none of the 8 assets )	263	28.7	117	26.6	146	30.6	

*Chi square test for independence or Fisher’s exact probability test for difference between men and women.

### Exposure to different forms of violence

#### Women

**Figure 1:**
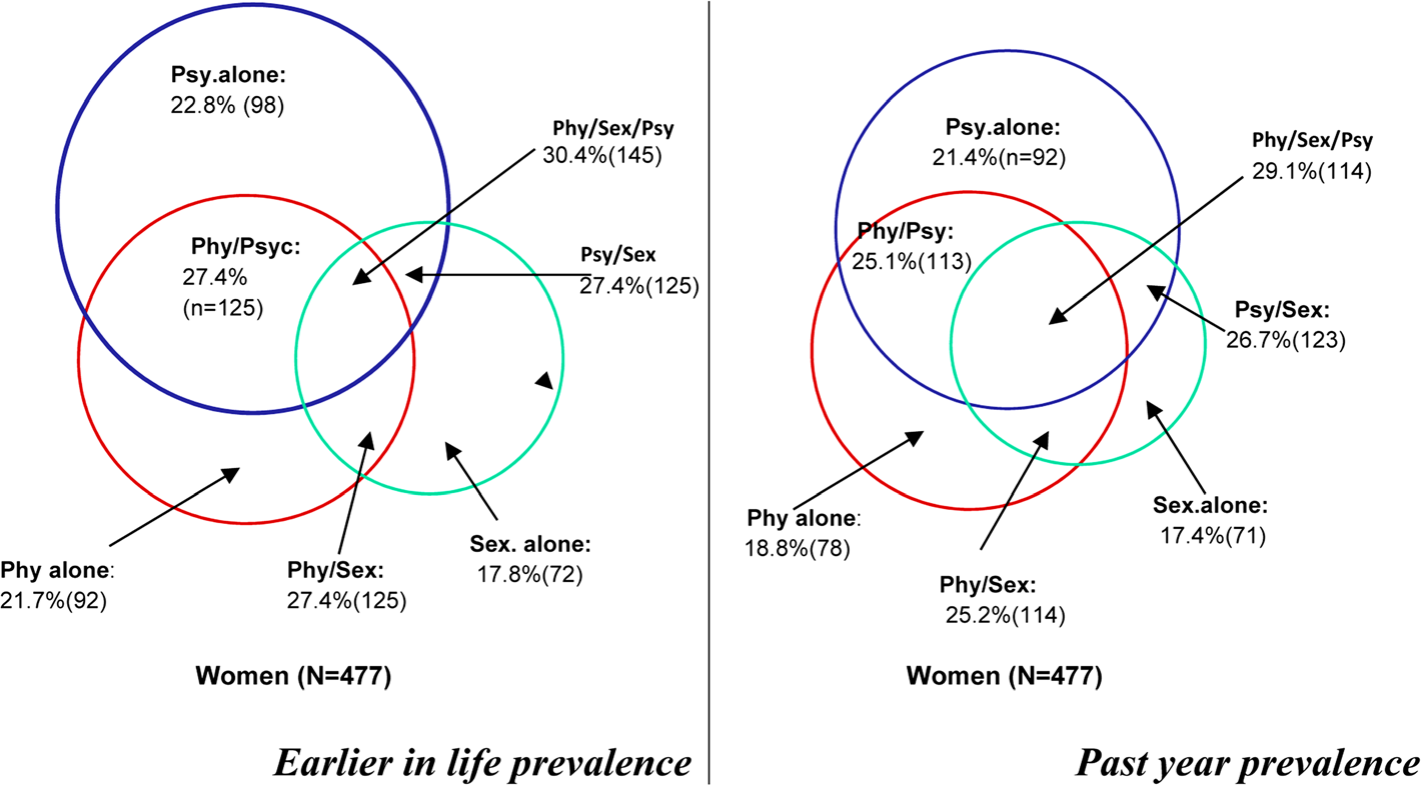
**Overlaps between different types of intimate partner violence for women (N = 477).** Phy: *Physical violence,* Sex: *Sexual violence,* Psy: *Psychological abuse.*

Of participating women, 21.7% (n = 92) had been subjected to *physical violence* earlier in life and 18.8% (n = 78) in the past 12 months; the moderate physical violence (slapped/threw something, pushed/showed) was more commonly observed than the severe physical violence (hit that could hurt, kicked/dragged or beaten, choked or burnt, threaten or used a weapon) as displayed in Table 3. For *sexual violence*, the corresponding figures were 17.8% (n = 72) and 17.4% (n = 71). The most commonly occurring form of violence in women was *psychological violence*, the prevalence being 22.8% for earlier in life and 21.4% for past year. In addition, the highest frequency of violence exposure (more than 3 times) was predominantly observed for earlier in life and past year estimates and for almost all acts included in the respective forms of violence (Table 3).When analysing the overlapping of the different forms of violence targeting women, the most commonly occurring form was the combination of all three forms of violence for earlier in life (30.4%) and for past year exposure (29.1%) (Figure 1).

**Table 3:** Prevalence and frequencies of earlier in life and past year physical, sexual and psychological violence experienced by women (N = 477)

	***Earlier in life***				***Past year***			
	**Violence exp. n (%)**	**Number of events**			**Violence exp. n (%)**	**Number of events**		
		**1**	**2 to 3**	**>3**		**1**	**2 to 3**	**>3**
**Physical violence** (N = 416)								
Slapped/threw something	84 (17.6)	21 (4.4)	23 (4.8)	40 (8.4)	69 (14.5)	25 (5.2)	16 (3.4)	28 (5.9)
Pushed/showed/pulled your hair	48 (10.1)	9 (1.9)	12 (2.5)	27 (5.7)	41 (8.6)	12 (2.5)	12 (2.5)	17 (3.6)
Hit that could hurt	52 (10.9)	10 (2.1)	13 (2.7)	29 (6.1)	47 (9.9)	13 (2.7)	14 (3.0)	20 (4.2)
Kicked/dragged or beating	48 (10.1)	8 (1.7)	17 (3.6)	23 (4.8)	40 (8.4)	10 (2.1)	13 (2.7)	17 (3.6)
Choked or burnt you on purpose	25 (5.2)	5 (1.0)	8 (1.7)	12 (2.5)	20 (4.2)	6 (1.3)	8 (1.7)	6 (1.3)
Threaten or used a weapon	20 (4.2)	3 (0.6)	8 (1.7)	9 (1.9)	17 (3.6)	5 (1.1)	6 (1.3)	6 (1.3)
*Summary measure of Physical violence*	*92 (21.7)*	*26 (5.5)*	*24 (5.0)*	*42 (8.8)*	*78 (18.8)*	*30 (6.3)*	*18 (3.8)*	*30 (6.3)*
**Sexual violence** (N = 409)								
Physically forced to have sexual intercourse	47 (9.9)	11 (2.3)	11 (2.3)	25 (5.3)	47 (9.9)	11 (2.3)	14(2.9)	22(4.6)
Did not want to have sexual intercourse	55 (11.6)	7 (1.5)	15 (3.2)	33 (6.9)	57 (12.0)	12(2.5)	21(4.4)	24(5.0)
Forced to do something sexual that felt degrading or humiliating	19 (4.0)	6 (1.3)	5 (1.0)	8 (1.7)	21 (4.4)	5(1.1)	10(1.1)	6(1.3)
*Summary measure of Sexual violence*	*72 (17.8)*	*15 (3.2)*	*15 (3.1)*	*42 (8.8)*	*71 (17.4)*	*15(3.1)*	*23(4.8)*	*33(6.9)*
**Psychological abuse** (N = 430)								
Insulted or made her feel bad about herself	69 (14.5)	9 (1.9)	16 (3.4)	44 (9.2)	62 (13.0)	11(2.3)	19(4.0)	32(6.7)
Belittled or humiliated her	60 (12.6)	10 (2.1)	17 (3.6)	33 (6.9)	55 (11.5)	11(2.3)	14(2.9)	30(6.3)
Did things to scare or intimidate her on purpose	75 (15.8)	11 (2.3)	13 (2.7)	51 (10.7)	73 (15.3)	15(3.1)	21(4.4)	37(7.8)
Threaten to hurt her or someone she cared about	27 (5.7)	6 (1.3)	6 (1.3)	15 (3.1)	24 (5.0)	5(1.1)	6(1.3)	13(2.7)
*Summary measure of Psychological abuse*	*98 (22.8)*	*11 (2.3)*	*19 (4.0)*	*68 (14.3)*	*92 (21.4)*	*14 (2.9)*	*25 (5.2)*	*53 (11.1)*

#### Men

Male respondents reported intimate partner violence exposure to a considerably lesser extent than women (Table 4). The frequency of the abuse was also less, i.e. one time was the most commonly reported frequency. However, results showed that only 4.0% (n = 17) were victims of physical violence earlier in life and 4.3% (n = 18) in past year (Table 4). Psychological abuse was the most commonly reported form of violence, indicating a past year estimate, which was greater than the earlier in life estimate (7.3% and 6.2%, respectively).

**Table 4:** Prevalence and frequency of earlier in life and past year violence in men (N = 440)

***Forms of violence***	***Earlier in life prevalence***	***Past year prevalence***
	**n (%)**	**n (%)**
**Physical violence** (N = 422)		
Slapped/threw something	12 (2.7)	10 (2.3)
Pushed/showed/pulled your hair	12 (2.7)	12 (2.7)
Hit that could hurt	13 (3.0)	7 (1.6)
Kicked/dragged or beating	9 (2.1)	4 (0.9)
Choked or burnt you on purpose	6 (1.4)	4 (0.9)
Threaten or used a weapon	6 (1.4)	5 (1.1)
*Summary measure of Physical violence*	*17 (4.0)*	*18 ( 4.3)*
**Sexual violence** (N = 410)		
Physically forced to have sexual intercourse	5 (1.1)	4 (0.9)
Did not want to have sexual intercourse	5 (1.1)	5 (1.1)
Forced to do something sexual that felt degrading or humiliating	9 (2.1)	4 (0.9)
*Summary measure of Sexual violence*	*10 (2.4)*	*6 (1.5)*
**Psychological violence** (N = 436)		
Insulted or made him feel bad about herself	18 (4.1)	17 (3.9)
Belittled or humiliated himr	19 (4.3)	20 (4.6)
Did things to scare or intimidate him on purpose	15 (3.4)	17 (3.9)
Threaten to hurt him or someone she cared about	13 (3.0)	13 (3.0)
*Summary measure of Psychological violence*	*27 (6.2)*	*32 (7.3)*

### Associations with socio-demographic and psycho-social factors

#### Women

In the crude logistic regression analyses, we found that having more than two children (OR 2.09; 1.27-3.45), resting with an incomplete primary education (OR 3.22; 1.57-6.62), without household assets (OR 1.81; 1.08-3.01) and with poor social support (2.94; 1.46-5.94) were statistically significant risk factors for women’s exposure to physical violence. For sexual violence against women, having more than two children (OR 1.72; 1.02-2.89) was a statistically significant risk factor. Psychological violence against women was associated with low educational attainment, related to the respondent (OR 1.93; 1.08-3.46) as well as to the partner (OR 1.94; 1.14-3.30) and poor social support (OR 2.63; 1.40-4.94) (Table 5).

**Table 5:** Associations between socio-demographic and psycho-social factors and women’s exposure to IPV, past year exposure

***Variables***	***Physical violence***		***Sexual violence***		***Psychological violence***	
	**n (%) with violence exp.**	**OR (95% CI)**	**n (%) with violence exp.**	**OR (95% CI)**	**n (%) with violence exp.**	**OR (95% CI)**
**1. Respondents age**						
20-29	46 (18.5)	1	41 (16.8)	1	54 (21.0)	1
30-35	32 (19.9)	1.10 (0.66-1.81 )	29 (18.4)	1.11 (0.66-1.88)	38 (22.8)	1.11 (0.69 -1.77)
**2. Number of children**						
0-2	39 (14.6)	1	39 (14.6)	1	54 (19.1)	1
> 2 or 3-11	39 (26.4)	**2.09(1.27 -3.45)**	32 (22.7)	**1.72 (1.02-2.89)**	38 (25.9)	1.47 (0.92-2.36)
**3. Respondent’s education**						
Complete primary, and above & vocational training	10 (8.3)	1	17 (13.4)	1	18 (14.1)	1
Incomplete primary	51 (22.7)	**3.22 (1.57 -6.62)**	36 (17.1)	1.34 (0.72-2.50)	55 (24.0)	**1.93 (1.08-3.46)**
**4. Partner’s education**						
Complete primary, and above & vocational training	24 (17.9)	1	26 (19.1)	1	25 (18.5	1
Incomplete primary	50 (26.9)	1.69 (0.97 -2.91)	41 (23.2)	1.28 (0.73-2.22)	60 (30.6)	**1.94 (1.14–3.30)**
**5. Assets in the household**						
Improved (at least one of 8 assets)	46 (15.9)	1	43 (15.0)	1	57 (18.9)	1
Poor (none of the 8 assets )	32 (25.4)	**1.81 (1.08-3.01 )**	28 (23.0)	1.69 (0.99-2.88)	35 (27.1)	1.59 (0.98-2.59)
**6. Social support**						
Good	10 (8.9)	1	14 (12.1)	1	13 (11.3)	1
Poor	68 (22.4)	**2.94 (1.46-5.94)**	57 (19.5)	1.76 (0.94-3.30)	79 (25.1)	**2.63 (1.40-4.94)**

Crude odds-ratios (OR) with their 95% confidence intervals (CI) (N = 477).

For physical violence, the multivariate logistic regression analysis was performed in four separate models and the risk factor pattern did not change as having more than two children (OR 2.05; 1.14-3.69), resting with an incomplete primary education (2.79; 1.33-5.84) and having low social support (2.40; 1.06-5.41) remained statistically significant in the final model. For psychological violence, only poor social support (2.61; 1.25-5.48) remained a statistically significant risk factor.

#### Men

As few men were exposed to physical or sexual violence from their partners, no statistically significant risk factor for physical and sexual violence were identified, therefore, no further analysis was performed for men in relation with exposure to physical and sexual violence. For psychological violence, however we found that low education, poor in household assets, with many children and a lower educated partner constituted risk factors for exposure to psychological violence in the bivariate analyses (Table 6), but none of them remained statistically significant risk factor in the multivariate analysis.

**Table 6:** Associations between socio-demographic and psycho-social factors and men’s exposure to IPV, past year exposure

***Variables***	***Physical violence***		***Psychological violence***	
	**n (%) with violence exp.**	**OR (95% CI)**	**n (%) with violence exp.**	**OR (95% CI)**
**1. Respondent’s Age**				
20-29	9 (3.2)	1	17 (5.8)	1
30-35	9 (6.6)	2.14 (0.83-5.52)	15 (10.5)	1.89 (0.91-3.90)
**2. Number of children**				
0-2	12 (3.4)	1	20 (5.6)	1
> 2 or 3-11	6 (8.6)	2.65 (0.96-7.31)	12 (15.8)	**3.18 (1.48-6.82)**
**3. Respondent’s education**				
Complete primary, and above & vocational training	4 (2.7)	1	6 (3.9)	1
Incomplete primary	12 (5.8)	2.24 (0.71-7.07)	23 (10.5)	**2.86 (1.13-7.19)**
**4. Partner’s education**				
Complete primary, and above & vocational training	3 (3.9)	1	5 (6.3)	1
Incomplete primary	14 (9.0)	2.45 (0.68-8.79)	26 (15.6)	**2.73 (1.01-7.40)**
**5. Assets in the household**				
Improved (at least one of 8 assets)	11 (3.5)	1	18 (5.6)	1
Poor (none of the 8 assets )	7 (6.4)	1.86 (0.70-4.92)	14 (12.0)	**2.27 (1.09-4.73)**
**6. Social support**				
Good	7 (4.0)	1	10 (5.6)	1
Poor	11 (4.5)	1.13 (0.42-2.98)	22 (8.6)	1.58 (0.73-3.42)

Crude odds-ratios (OR) with their 95% confidence intervals (CI) (N = 440).

## Discussion

In this study from rural and urban parts of Southern Province of Rwanda, we found that both men and women were exposed to intimate partner violence although men to a considerably lesser extent than women. IPV exposure, in the form of repeated acts of physical, sexual and psychological violence, was commonly faced by women, while men reported only single events of violence. Women with low educational attainment and living in poor life circumstances were most exposed to each of the forms of violence.

### Intimate partner violence against women

Rwanda is currently seen as a country with a relatively high level of gender equality at legal and policy level, and a gender-based violence (GBV) intolerance reflected in the law on GBV mentioned above [16]. Even so, violence against women in intimate partnerships remains a public health issue sustained by cultural norms, reflected as a gender power imbalance in relationships [26]. The general belief is that IPV is a purely domestic issue [27,28] that is not of societal concern, and therefore seldom disclosed outside the household. Equally, when IPV is disclosed, other factors such as women’s economic dependence on their husbands support IPV against women. Furthermore, when there is a known case of IPV, the Rwandan culture first suggests application of a community dialogue (named “Gacaca”); i.e. family or local leaders approach the couple, with reconciliation as the goal and divorce as the final alternative.

The 2010 Rwanda Demographic and Health Survey (DHS) results on domestic violence against women aged 15–49 years shows that the proportion of women who exclusively experienced lifetime physical violence is about 26% [29], which could possibly be compared with earlier in life estimates from the present study (21.7%). Findings from a Ugandan study for women’s exposure to past year physical violence (20%) is close to our finding (18.8%) [11].

As for past year occurrence of sexual violence, the prevalence in our study (17.4%) was quite similar to the Tanzanian study prevalence (18.3%) [30], but somewhat higher than what has been observed among Rwandan adolescents attending school (15.5%) [31] and considerably lower than the Ethiopian study prevalence (44.4%) [30].

Recent findings from two studies in high income countries on men’s and women’s exposure show that the occurrence of physical and psychological violence are of the same magnitude for men and women, while considerably more women are exposed to sexual abuse [3,32]. Other studies find all forms of IPV exposure to be more common among women than men [11,33].

Although findings in the present study show that women are more exposed to IPV than men, it is important to highlight that there is a possibility of underreporting of violence exposure among women, due to the fear of revenge from the partner [34]. Other possible reasons for women’s underreporting of partner violence exposure include the humiliation, the shame of being victim of IPV and the wish to stay in the relationship [35].

### Intimate partner violence against men

In our study, men were to a considerably lesser extent exposed than women to any of the forms of violence investigated. Psychological IPV was the most common form of IPV targeting men, as is the case in the study by Fawole et al. from Nigeria [36]. From the neighbouring Uganda, considerably more men report lifetime exposure to physical violence from their female partner [7] than what is commonly seen in such studies.

An interesting reflection is whether our observed discrepancy in reporting of IPV incidents between men and women is due to men’s denial of incidents or to a gender power imbalance, i.e. attributed to the general subordination of women to men in the society. It is well known that power is a central aspect of gender relations and women have less access to most kinds of power, and most stereotypic male–female differences result from this imbalance [37]. Denial of incidents could be at hand due to men’s hesitation to report any violence or abuse exposure from the wife/partner, as this would be in sharp contrast to accepted gender norms [38]. Another explanation for possible denial could be men’s general neglect of violence inflicted by a woman, instead it is belittled and considered ridiculous and insignificant by exposed men and therefore not reported [39]. A final explanation could be women’s physical disadvantage, making any act of violence less threatening for a physically stronger male person and hereby subject to denial of incidents over time [40,41]. On the other hand, the discrepancy in reporting may well reflect a situation where men are to a considerably lesser extent exposed to IPV. Support for this assumption is given in several studies from Sub-Saharan African countries including Rwanda, that report on large proportions of men and women with supportive attitudes towards wife beating [13]. This reflects traditional gender norms based on a substantial gender power imbalance [42].

### Factors associated with IPV

Our results illustrate that poor life circumstances with no assets in the household and many children in the family were associated with physical violence. This can be linked to financial stress, reflecting difficulties in handling everyday life. Such a stressful condition may result in miscommunication within couples and abuse towards women by men who are seen as key wage earners [43,44]. Moreover, educational attainment, which is usually considered as a door to opportunities was generally poor in our study population and highly associated with physical and psychological violence. Studies from various countries including 17 countries in Africa, Sri-Lanka, Haiti and Nepal show that low educational status constitute a risk factor for IPV directed at women [45,46]. For women, good social support is protective against physical violence [47] by its potential to empower women [48]. Social support especially from family members makes women feel more secure with some protection.

### Methodological considerations

In this study, the past year violence estimates were used in the analyses as they are frequently assumed to be more precise measures of IPV than earlier in life estimates due to lower recall bias [24]. However, as people believe culturally that family matters and particularly violence exposure should not be disclosed to others, there is the possibility of general underreporting. Nevertheless, the data collection procedure in this study was performed with great care, by experienced and trained clinical psychologists who were able to establish a good discussion climate in Kinyarwanda with the individual participants. Furthermore, the data collectors were of the same sex and of similar age to the participants, which has been shown to improve the accuracy of the reporting in interviews [49]. The data is hereby considered to be of high internal and external validity with possibly some underreporting but with high precision and objectivity in the interview situation. As this was a cross-sectional study, only statistically significant associations with all forms of violence are given, and no causal relationship can be established. In addition, the comparison of our findings to other studies needs to be done with care given the narrow age span (20–35 years) of the current study sample.

## Conclusion

In Rwanda, intimate partner violence is most commonly exercised towards women while men’s exposure to IPV seems to be considerably lower. This might be due to recall bias and denial of incidents in men but is certainly also explained by gender power imbalance, reinforced by cultural norms and society’s tolerance to traditional gender norms.

Income generating activities as well as access to financial credits, combined with educational awareness on gender equality and human rights issues would contribute to women’s empowerment. Promotion of gender equality at individual level is needed to make a positive difference in a relatively short term. The media needs to be involved to produce public debates on intimate partner violence, condemning such abuses and revealing their important public health consequences for the woman and her family. The health sector with the help of the Rwandan police carries the responsibility to identify those exposed to partner violence and be able to offer an adequate treatment and support.

In this setting, qualitative studies are needed to improve the knowledge and understanding of men’s and women’s exposure to IPV to shed light on motives, seriousness and level of threat that such violence induces in the victim. This would in turn give guiding principles for sex-specific preventive and intervention strategies.

## Abbreviations

IPV: Intimate partner violence; WHO: World health organization; GBV: Gender based violence; RwMHBC project: The rwanda violence, mental health and barriers to care project; SPH: School of public health; RNEC: Rwanda national ethic committee; NISR: National institute of statistics of rwanda; DHS: Demographic and health survey.

## Competing interest

The authors declare that they have no competing interests.

## Authors’ contributions

GK designed the study. The study questionnaire was developed by GK, JN and IM, and AU complemented it. GK and IM further assisted AU in the statistical analyses and contributed to the manuscript. AU developed the study methodology, coordinated the data collection activities, performed all the statistical analyses and drafted the manuscript. JN assisted in the planning and in the writing. All authors read and approved the final manuscript.

## Pre-publication history

The pre-publication history for this paper can be accessed here:

http://www.biomedcentral.com/1472-6874/14/99/prepub

## Supplementary Material

Additional file 1 STROBE Statement—Checklist of items that should be included for cross-sectional studies. Click here for file
